# Bacteriological Profiles and Antimicrobial Susceptibility Patterns Among Isolates in Tonsillitis Patients Attending a Tertiary Care Hospital

**DOI:** 10.7759/cureus.72403

**Published:** 2024-10-25

**Authors:** Vaishnavi B Shevale, Ravindra V Shinde, Satish R Patil

**Affiliations:** 1 Department of Microbiology, Krishna Institute of Medical Sciences, Krishna Vishwa Vidyapeeth (Deemed to be University), Karad, IND

**Keywords:** antimicrobial susceptibility, clinical and laboratory standards institute, gram-negative organisms, gram-positive organisms, tonsillitis

## Abstract

Background: Tonsillitis, primarily affecting children, is an inflammation of the pharyngeal tonsils caused by bacterial pathogens such as *Staphylococcus aureus* and *Streptococcus pyogenes* or viral agents. It can be classified as acute, chronic, or recurrent based on episode duration and frequency. Effective treatment requires identifying causative pathogens and assessing antibiotic resistance patterns. This study aims to update the bacterial isolates and antibiotic resistance of tonsillitis pathogens to support empirical treatment strategies and inform infection control policies.

Objective: This research was undertaken to assess the bacterial profiles and antibiotic susceptibility patterns of isolates obtained from tonsillitis patients.

Materials and methods: This research was undertaken in the Department of Microbiology at Krishna Institute of Medical Sciences, Krishna Hospital and Medical Research Centre, Karad, District Satara, during the period from November 2022 to November 2023. Specimen collection and processing were performed following standard guidelines. Tonsillar swabs were collected and processed for pathogen isolation and identification using Gram staining and various biochemical tests; the susceptibility to antimicrobials was tested using the Kirby-Bauer disc diffusion method on Mueller-Hinton agar in accordance with Clinical and Laboratory Standards Institute 2022 recommendations.

Result: Out of 105 clinically confirmed tonsillitis cases, 93 (88.57%) were culture-positive, with three cases presenting two isolates each, resulting in a total of 96 isolates, and 12 cases (11.43%) were culture-negative. A greater prevalence was noted among male patients (56 (60.21%)), with the highest occurrence observed in the 0-20-year age group (60 (64.51%)). Among the 96 isolates, 52 (54.16%) were Gram-negative and 44 (45.83%) were Gram-positive. The predominant isolate identified was *S. aureus* (39 (40.62%)), followed by *Pseudomonas aeruginosa *(19 (19.79%)). Testing for antimicrobial susceptibility revealed that *S. aureus* was the most common isolate, showing sensitivity to chloramphenicol in 38 cases (97.43%), while *P. aeruginosa, *the second most common isolate*, *showed the highest sensitivity to azithromycin in 17 cases (89.47%).

Conclusion: Among 105 clinically confirmed tonsillitis cases, 93 were culture-positive, resulting in 96 isolates. *S. aureus *was the organism found most frequently, which showed maximum sensitivity to chloramphenicol, followed by *P. aeruginosa*, which is sensitive to azithromycin. Azithromycin, a macrolide, and chloramphenicol, a broad-spectrum antibiotic, both block bacterial protein synthesis via various mechanisms. This emphasizes the critical importance of analyzing bacterial profiles and resistance patterns to guide antimicrobial stewardship in tonsillitis management.

## Introduction

The term "tonsillitis" describes an inflammation of the pharyngeal tonsils, which primarily affects youngsters. It is an inflammatory condition that is typically caused by infection in the tonsillar tissue. The primary locations for humoral and cell-mediated immune responses are the tonsils [1]. From birth, the upper respiratory tract's typical flora is established [2]. The ratio of anaerobic to aerobic bacteria varies with the concentration of oxygen throughout the oral cavity [2]. Since the tonsils lack sensorial lymphatics, the specialized epithelium plays an important role in antigen presentation and processing. The bacteria most frequently isolated include *Staphylococcus aureus *and *Streptococcus pyogenes* [1,3].

The tonsils, which grow at 6.5 weeks between the second and third arches ventrally, are made of lymphatic tissue and are a part of Waldeyer's ring together with the lingual tonsil and adenoid tubal. By being the first line of defense against irritants, they play a crucial role in protecting the body from inhaled or ingested microorganisms. The lateral oropharynx contains the palatine or faucial tonsils. They can be located between the palatoglossal arch and the palatopharyngeal arch. A wealth of data indicates that the tonsils have a role in secretory immunoglobulin production [2]. The specific function of the tonsils is to transfer foreign materials directly from the outside to the lymphoid cells. Human tonsils are immunologically active from four to 10 years of age [4]. Following puberty, the tonsils undergo involution, leading to an altered ratio of T cells to B cells [2].

The tonsils house T lymphocytes, macrophages, and germinal centers of B lymphocytes because they aid in immunological acquisition and defense through the presentation of antigens. They are the human mucosa-associated lymphoid tissue (MALT) system's initial and most accessible station [5]. Due to its contagious nature, tonsillitis can be extensively classified into three categories, depending on the frequency and duration of the associated symptoms.

Acute tonsillitis

This category includes situations in which the illness lasts for three days to about two weeks. An upper respiratory tract illness known as acute tonsillitis is linked to inflammation of the palatine tonsillar lymphoid tissue [4]. Acute tonsillitis is a type of infection that lasts for a short while. Because the tonsillar rings' lymphoid tissue matures and completes after that age, acute tonsillitis is uncommon in children under the age of one to one and a half. It is challenging to adopt timely laboratory diagnostics for diseases like acute tonsillitis, which can be caused by many species, particularly in countries with limited resources. Acute suppurative tonsillitis is most frequently caused by group A and B hemolytic streptococci [4].

Chronic tonsillitis

Chronic tonsillitis cases are defined by symptoms persisting for three weeks. The most prevalent illness of the throat, chronic tonsillitis, primarily affects younger people. Mostly, a physical examination and history are used to diagnose this illness [6].

Recurrent tonsillitis

Recurrent tonsillitis is commonly defined, though somewhat arbitrarily, as the occurrence of five or more episodes of tonsillitis within a one-year period [7]. A related condition, peritonsillar abscess, is more common in adults and teenagers, arising when an infection spreads beyond the tonsils. Data from developing nations show regional variations in antibiotic resistance patterns, with tonsillitis-causing bacteria constantly evolving. This study aims to guide empirical antibiotic selection by analyzing isolated pathogens and their antibiotic susceptibility, improving treatment outcomes and reducing time and costs [3].

## Materials and methods

Study design and sample size

The present observational-cross-sectional study was conducted in the Department of Microbiology, Krishna Institute of Medical Sciences, Krishna Hospital and Medical Research Centre. Ethical approval (approval number KIMSDU/IEC/04/2022) was secured from the ethical committee, Krishna Institute of Medical Sciences (Deemed to be University), Karad, over the duration from November 2022 to November 2023 under protocol number 070/2021-2022.

According to the study conducted by Ughasoro et al. [4], from the Department of Pediatrics at the University of Nigeria, Enugu Campus, the sample size for the present study was calculated using the formula n = 4pq/l^2^. In this formula, p represents the prevalence rate (15.3), q is derived from 100 - P (100 - 15.3 = 84.7), and l denotes the allowable error (7). Based on these parameters, the sample size was calculated as 4 × 15.3 × 84.7/7^2^ = 105. Therefore, a total of 105 samples were included in the study.

Inclusion criteria

Patients of all age groups and both genders who attended the hospital and provided informed consent to participate in the study were included.

Exclusion criteria

Individuals who were either unwilling to participate or not present at the selected sites during data collection were excluded.

Sample collection and processing

Specimens were collected by a trained Senior Resident and Clinician in the Ear, Nose, and Throat (ENT) department. The oral cavity of the patient was opened widely and illuminated adequately with a headlamp, and the tongue was depressed with a sterile wooden specular. The sterile cotton wool swab stick was rubbed firmly on the surfaces of the inflamed tonsils, avoiding contact with the tongue or buccal mucosa. Two swabs were obtained from the same locations and were sent to the Microbiology department for further processing. One was utilized to prepare a smear, while the other was designated for culture. The specimens were processed within a two-hour period following collection. Gram staining was performed to prepare the smear. The specimens were inoculated onto nutrient agar, blood agar, and MacConkey's agar media. The plates were incubated for 24 hours at 37°C. The identification of growth was conducted through colony morphology assessment, Gram staining, and appropriate biochemical tests [8].

Antimicrobial susceptibility testing

Antimicrobial susceptibility testing of the isolates was carried out using the Kirby-Bauer disc diffusion method on Mueller-Hinton agar, adhering to the Clinical and Laboratory Standards Institute (CLSI) [9] guidelines with commercial discs. Discs of antibiotics in the antibiogram included ciprofloxacin (5 µg), ofloxacin (5 µg), cefuroxime (30 µg), cefotaxime (30 µg), ceftriaxone (30 µg), azithromycin (15 µg), ampicillin (10 µg), levofloxacin (5 µg), norfloxacin (10 µg), gentamicin (10 µg), chloramphenicol (30 µg), doxycycline (30 µg), linezolid (30 µg), amoxicillin-clavulanic acid (20 µg/10 µg), cefixime (5 µg), piperacillin (100 µg), piperacillin-tazobactam (30 µg), and cefoperazone (75 µg).

## Results

Out of 105 tonsillitis cases, 93 (88.57%) were culture-positive and 12 (11.43%) were culture-negative. Out of 105 cases of tonsillitis, 83 (79.05%) had an acute type and 22 (20.95%) had a chronic type.

Among the 105 clinically diagnosed tonsillitis cases, 93 were confirmed as culture-positive, with three cases yielding two isolates each, resulting in a total of 96 isolates. In the age range of 0-20, a total of 60 (64.51%) were culture-positive cases; among these, 42 individuals (45.16%) were men and 18 (19.35%) were women. Within the age range of 21-40, overall, there were 27 culture-positive cases (29.02%), including 12 (12.90%) male patients and 15 (16.12%) female patients. Six cases (6.47%) were identified as culture-positive within the age bracket of 41-60, comprising two male patients (2.15%) and four female patients (4.30%) (Table 1).

**Table 1:** Age- and gender-wise distribution of tonsillitis cases n: number

Age range	Male patients, n (%)	Female patients, n (%)	Total, n (%)
0-20	42 (45.16)	18 (19.35)	60 (64.51)
21-40	12 (12.90)	15 (16.14)	27 (29.02)
41-60	2 (2.15)	4 (4.30)	6 (6.47)
≥60	0 (0)	0 (0)	0 (0)
Total	56 (60.21)	37 (39.79)	93 (100)

In the current study, the majority of isolates were Gram-negative, accounting for 52 (54.16%), compared to Gram-positive isolates, which were 44 (45.83%). A total of 96 isolates were obtained from 93 culture-positive cases, with three cases each producing two isolates in patients diagnosed with tonsillitis (Table 2).

**Table 2:** Distribution of bacterial isolates among tonsillitis cases n: number

Gram-positive isolates (n = 44)	Total, n (%)
Staphylococcus aureus	39 (40.62)
Streptococcus pyogenes	5 (5.2)
Gram-negative isolates (n = 52 )	Total, n (%)
Escherichia coli	13 (13.54)
Klebsiella pneumoniae	9 (9.37)
Klebsiella aerogenes	6 (6.24)
Enterobacter aerogenes	1 (1.07)
Pseudomonas aeruginosa	19 (19.79)
Acinetobacter baumannii	4 (4.17)
Total	96 (100)

The bacterial isolates were evaluated for their susceptibility to antimicrobial agents, and their sensitivity patterns were observed. *S. aureus* emerged as the predominant pathogen among the Gram-positive isolates.

**Figure 1:**
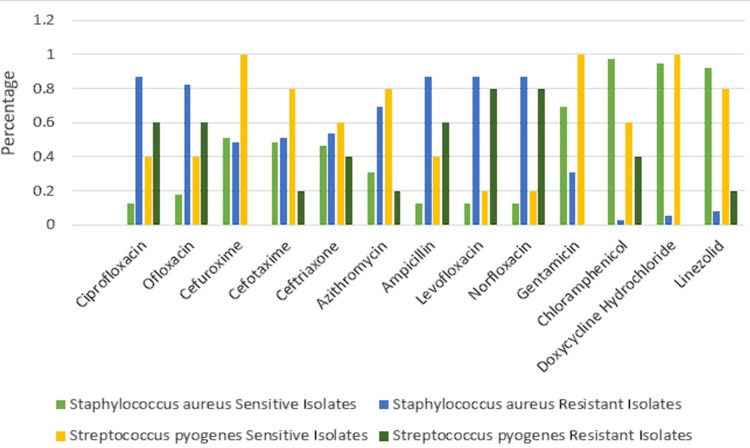
Antimicrobial susceptibility pattern of Gram-positive bacteria

*S. aureus* revealed the highest sensitivity to chloramphenicol (38 (97.43%)) along with doxycycline hydrochloride (21 (53.84%)), gentamicin (27 (69.23%)), and linezolid (36 (92.30%)), whereas maximum resistance was to ciprofloxacin, levofloxacin, and norfloxacin (34 (87.17%)) followed by ampicillin (34 (87.17%)) and ofloxacin (32 (82.05%)). *S. pyogenes* showed maximum sensitivity to cefuroxime, gentamicin, and doxycycline hydrochloride (5 (100%)) followed by cefotaxime, azithromycin, and linezolid (4 (80%)). *S. pyogenes* showed maximum resistance to levofloxacin and norfloxacin (4 (80%)) followed by ciprofloxacin, ofloxacin, and ampicillin (3 (60%)) (Figure 1).

**Figure 2:**
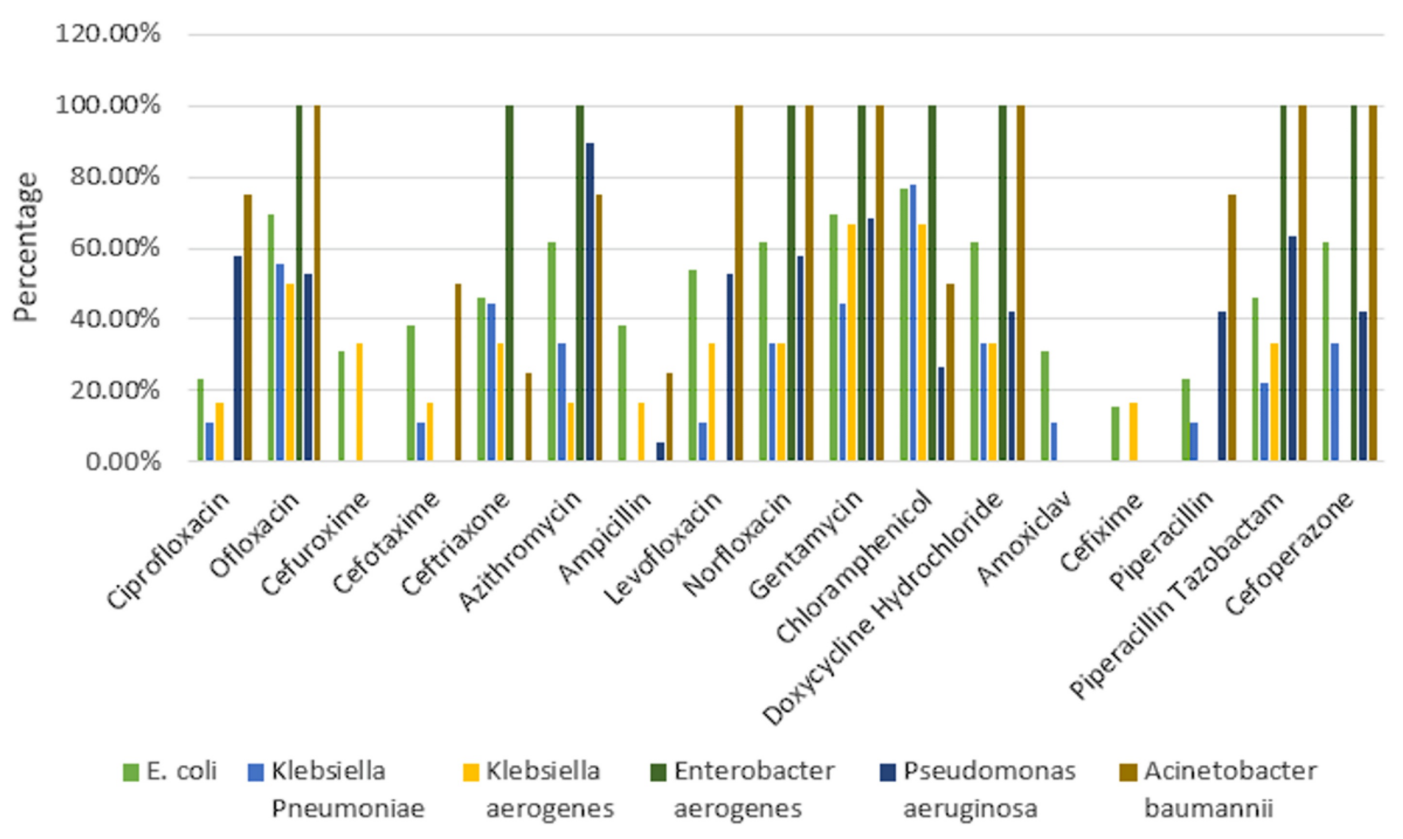
Antimicrobial sensitivity pattern of Gram-negative bacteria E. coli : *Escherichia coli*

**Figure 3:**
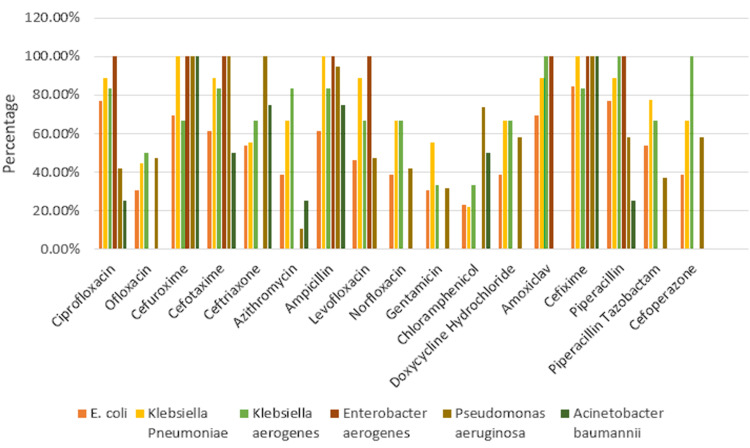
Antimicrobial resistance pattern of Gram-negative bacteria E. coli: *Escherichia coli*

*Escherichia coli* exhibited the highest sensitivity to chloramphenicol (10 (76.92%)) followed by ofloxacin and gentamicin (9 (69.23%)) and higher resistance to cefixime (11 (84.61%)) along with ciprofloxacin and piperacillin (10 (76.92%)). *Klebsiella pneumoniae* exhibited the highest sensitivity to chloramphenicol (7 (77.77%)) followed by ofloxacin (5 (55.55%)). *K. pneumoniae* showed resistance to cefuroxime, ampicillin, and cefixime (9 (100%)) followed by ciprofloxacin, cefotaxime, levofloxacin, amoxiclav, and piperacillin (8 (88.88%)). *Klebsiella aerogenes* showed maximum sensitivity to gentamicin and chloramphenicol (4 (66.66%)), followed by ofloxacin (3 (50%)), and maximum resistance to amoxiclav, piperacillin, and cefoperazone (6 (100%)). *Enterobacter aerogenes* showed maximum sensitivity to ofloxacin, ceftriaxone, azithromycin, norfloxacin, gentamicin, chloramphenicol, doxycycline hydrochloride, piperacillin-tazobactam, and cefoperazone (1 (100%)) and showed maximum resistance to ciprofloxacin, cefuroxime, cefotaxime, ampicillin, levofloxacin, amoxiclav, cefixime, and piperacillin (1 (100%)). *Pseudomonas aeruginosa* revealed higher sensitivity to azithromycin (17 (89.47%)) along with gentamicin (13 (68.42%)). *P. aeruginosa* exhibited the highest resistance to cefuroxime, cefotaxime, ceftriaxone, and cefixime (19 (100%)) followed by ampicillin (18 (94.73%)). *Acinetobacter baumannii* revealed the highest sensitivity to ofloxacin, levofloxacin, norfloxacin, gentamicin, piperacillin-tazobactam, and cefoperazone (4 (100%)) and maximum resistance to cefuroxime and cefixime (4 (100%)) followed by ceftriaxone and ampicillin (3 (75%)) (Figures 2, 3).

## Discussion

The current study found that 93 out of 105 tonsillitis cases (88.57%) were culture-positive, while 12 cases (11.43%) were culture-negative. In comparison, Manikandan and Amsath [10] reported that 93.6% (337 out of 360 cases) were culture-positive, and 9.3% (23 cases) were culture-negative. In contrast, Bakir and Ali [11] reported a high prevalence of culture-positive cases, with 67% (134 out of 200 cases) testing positive, which is lower than the present study, while 33% (66 cases) were culture-negative. Furthermore, Bunyan et al. [12] documented that 93.1% (190 out of 204 cases) were culture-positive, and 6.9% (14 cases) were culture-negative, showing a slightly higher percentage of culture-positive cases compared to the present study. These observations indicate that although the proportion of culture-positive tonsillitis cases in the present study is high, it is somewhat lower compared to other studies, particularly those by Manikandan and Amsath and Bunyan et al., which reported slightly higher culture-positivity rates.

The present study revealed that 79.05% (83 out of 105) of tonsillitis cases were acute, showing a considerably greater rate than the 20.95% (22 cases) of chronic tonsillitis observed. In contrast, Darod et al. [13] reported a predominance of acute tonsillitis cases, with 54% (202 out of 254 cases) being acute, compared to 13.9% (52 cases) that were chronic. Although both studies found a higher prevalence of acute tonsillitis, the proportion of acute cases in the current study was markedly greater than that observed in Darod et al., indicating a stronger predominance of acute tonsillitis in the current study population. Overall, the higher percentage of acute cases observed in both studies highlights the consistent observation that acute tonsillitis occurs more frequently than chronic tonsillitis.

The present study found that 60.21% of tonsillitis cases were male patients, while 39.79% were female patients, out of a total of 93 cases. Purohit [14] also documented a higher prevalence of male patients, with 58.5% of 200 tonsillitis cases occurring in male patients and 41.5% in female patients, reflecting a slightly lower proportion of affected male patients compared to the present study. In comparison, Prajapati et al. [15] reported that 56.3% of the 110 tonsillitis cases were male patients, with 43.7% female patients, indicating a marginally higher prevalence of male cases in the present study. Furthermore, Vijayashree et al. [16] observed that 55% of the 100 participants were male patients, and 45% were female patients, showing a slightly lower proportion of male cases compared to the present study. Hathal et al. [17] found that 55% of 92 tonsillitis cases were male patients and 45% were female patients. This aligns with the findings of the other studies, demonstrating a male predominance. However, the proportion of male cases in the current study is marginally greater than in these studies, indicating variability in the gender distribution of tonsillitis across different populations.

In the present study, 60.90% of the 105 tonsillitis cases were in the 0-20 age group, which is consistent with findings from Karim et al. [18] where 62.5% (100 out of 160 cases) of tonsillitis cases were in this age group. Al-Tameemi et al. [19] reported an even higher percentage in the 0-20 age group, with 87.87% (29 out of 33 cases) of tonsillitis cases, indicating a greater prevalence of younger patients compared to the present study. In comparison, Raju and Selvam [20] found that 71% (109 out of 149 cases) of tonsillitis cases occurred in the 0-20 age group, supporting the findings observed in the current study. Conversely, Abraham et al. [1] showed a lower percentage of tonsillitis cases in the 0-20 age group, with only 43.1% (209 out of 485 cases) falling within this category, which is considerably lower than the percentages reported in the current study. These comparisons underscore the generally higher prevalence of tonsillitis in the younger age category, including variation across different studies.

The present study identified that 54.16% (52 out of 96) of the isolates were Gram-negative, while 45.83% (44 out of 96) were Gram-positive. In comparison, Kumar et al. [21] found that 45.18% (712 out of 1,268) of the isolates were Gram-negative and 42.39% (668 out of 1,268) were Gram-positive. Although the percentage of Gram-negative isolates in their study was lower than that observed in the current study, the absolute numbers indicate a higher overall rate of both Gram-negative and Gram-positive isolates. Conversely, Purohit [14] documented a significantly higher proportion of Gram-negative isolates, with 70.5% (141 out of 200 cases), compared to 21.0% (42 out of 200 cases) for Gram-positive isolates. This indicates a more pronounced predominance of Gram-negative bacteria in their study compared to the present study. Anand [22] reported that 67% (134 out of 200) of the isolates were Gram-negative, while 22% (44 out of 200) were Gram-positive. This study also reflects a higher percentage of Gram-negative isolates contrasted with the present study. Therefore, the current study shows a higher prevalence of Gram-negative isolates compared to Gram-positive ones; the studies by Purohit and Anand demonstrate an even greater predominance of Gram-negative bacteria, indicating higher Gram-negative isolation, which contrasted with the present study.

In the current study, *S. aureus* was the most commonly isolated organism, representing 40.62% (39 out of 96) of the isolates. This was proceeded by *P. aeruginosa* at 19.79% (19 out of 96), *E. coli* at 13.54% (13 out of 96), and *K. pneumoniae* at 9.37% (nine out of 96). In comparison, Aktar et al. [23] reported a similar pattern of isolation among the organisms. Out of 61 isolates, *S. aureus* was found in 42.62% (26 out of 61), *P. aeruginosa* in 26.23% (16 out of 61), *E. coli* in 19.67% (12 out of 61), and *K. pneumoniae* in 9.84% (six out of 61). Both studies demonstrated a similar distribution of pathogens, with *S. aureus* being the most prevalent organism, followed by *P. aeruginosa*, *E. coli*, and *K. pneumoniae*. The proportions of isolated organisms reported by Aktar et al. correspond with those observed in the present study, indicating a consistent and analogous pattern of bacterial distribution in tonsillitis cases.

In the current study, *P. aeruginosa* exhibited a higher sensitivity toward azithromycin; similar findings were documented by Sadoh et al. [24]. Also, greater resistance was noted against amoxicillin-clavulanate (amoxiclav) among most Gram-negative organisms like *K. pneumoniae*, *K. aerogenes*, and *E. aerogenes*, which correlates with the findings published by Purohit [14].

This study was restricted to bacteriological analysis and left out viral or fungal pathogens. Additionally, bacterial drug resistance was assessed using phenotypic methods rather than genotypic approaches. Researchers interested in further exploring drug resistance mechanisms through genotypic methods are encouraged to build upon the observations of the present study to enhance their understanding of resistance patterns.

## Conclusions

Respiratory tract infections (RTIs), particularly tonsillitis, pose a global health challenge due to rising antibiotic resistance driven by inappropriate antibiotic use and bacterial beta-lactamase production. A huge amount of antimicrobials being prescribed for throat-related illness may often be unnecessary many a time and may exacerbate this issue, increasing morbidity and mortality, particularly in developing countries. The careful selection of antibiotics within treatment regimens is critical to curbing this trend, making sure that drugs are used only when appropriate. Understanding bacterial resistance patterns is essential for informed antimicrobial stewardship. Chloramphenicol, a broad-spectrum bacteriostatic antibiotic, disrupts bacterial protein synthesis, while azithromycin, a newer macrolide, targets bacterial ribosomes and enhances antibiotic efflux, offering alternative treatment options amid escalating resistance. In the present study, these antibiotics are explored as potential treatment options to address the growing challenge of resistance.
